# Image Encryption Algorithm Based on the H-Fractal and Dynamic Self-Invertible Matrix

**DOI:** 10.1155/2019/9524080

**Published:** 2019-06-13

**Authors:** Xuncai Zhang, Lingfei Wang, Ying Niu, Guangzhao Cui, Shengtao Geng

**Affiliations:** School of Electrics and Information Engineering, Zhengzhou University of Light Industry, Zhengzhou 450002, China

## Abstract

In this paper, an image encryption algorithm based on the H-fractal and dynamic self-invertible matrix is proposed. The H-fractal diffusion encryption method is firstly used in this encryption algorithm. This method crosses the pixels at both ends of the H-fractal, and it can enrich the means of pixel diffusion. The encryption algorithm we propose uses the Lorenz hyperchaotic system to generate pseudorandom sequences for pixel location scrambling and self-invertible matrix construction to scramble and diffuse images. To link the cipher image with the original image, the initial values of the Lorenz hyperchaotic system are determined using the original image, and it can enhance the security of the encryption algorithm. The security analysis shows that this algorithm is easy to implement. It has a large key space and strong key sensitivity and can effectively resist plaintext attacks.

## 1. Introduction

In modern society, technologies such as the Internet and block-chains are rapidly developing, and human beings have entered the big data era. Internet technology has brought great convenience to human life and promoted the establishment of global information access. With the development of multimedia technology, digital offices and electronic payments have become more popular in various fields of human life. Compared with textual information, the informative features that are expressed by images are more intuitive, and the amount of information that images contain has increased. At this stage, images are being used as the main carrier of information. While enjoying the convenience brought by the information society, we must also be more vigilant about the disasters that can be caused by information leakage. For example, in June 2013, former CIA employee Snowden revealed the “PRISM Project” to the world. Some high-tech companies with great influence left back doors in the equipment that they produced, making it convenient for the US government to monitor the public. During the Korean Winter Olympics in January 2018, the identity information and bank account information of a large number of athletes and spectators were maliciously acquired by hackers, thereby causing adverse effects. Protecting the security of information and avoiding losses due to information leakage is an urgent task for human beings. Traditional encryption algorithms such as DES [1–3] and RSA [4–9] have a wide range of applications in text encryption, but the applications of traditional encryption algorithms are not sufficient to meet the timeliness and security requirements of image information encryption. Therefore, how to encrypt image information quickly and effectively has become a popular research field.

There are two main types of methods in image encryption algorithms: scrambling [10–15] and diffusion [16–20]. Scrambling is achieved by transforming the positions of the pixels. Transforming the positions of the pixels can decrease the correlation between adjacent pixels and achieve encryption. For example, in 2004, Maniccam et al. proposed an encryption method based on SCAN mode in which the image is encrypted by using a different scanning path [21]. In 2010, Jolfaei et al. proposed an encryption algorithm based on the Henon chaotic system that uses the sorting transformation method to encrypt images. This method makes cipher images more pseudorandom [22]. In 2011, Zhu proposed an encryption method based on bit-plane scrambling [10]. Diffusion is performed by changing the values of the pixels. Diffusion encryption can enhance the randomness and break the statistical characteristics of the cipher images. For example, in 2009, Acharya proposed an encryption algorithm based on the Hill matrix that uses an invertible matrix to encrypt images [23]. In 2011, El-Zoghdy proposed an improved DES algorithm to encrypt images [24]. In recent years, some hybrid image encryption algorithms have been proposed. For example, in 2004, Gehani proposed an encryption method using DNA strings that applied DNA coding to image encryption [25]. In 2005, Guan proposed an encryption algorithm based on Arnold-Chen chaotic sequences that combined scrambling and diffusion in the image encryption process [26]. In 2008, Tong et al. proposed an encryption method that combined cyclic shifts and sequence encryption [27].

In this paper, an image encryption algorithm based on the H-fractal structure and dynamic self-invertible matrix is proposed. This algorithm combines the scrambling and diffusion encryption methods.  Section 2 introduces the basic theory of this algorithm, Section 3 introduces the encryption scheme, and the security analyses of this encryption algorithm are given in Section 4. The results of the security analysis show that the encryption algorithm has good security, and it can be applied in the field of image encryption.

## 2. Fundamental Theory

### 2.1. Lorenz Hyperchaotic System

**Figure 1:**
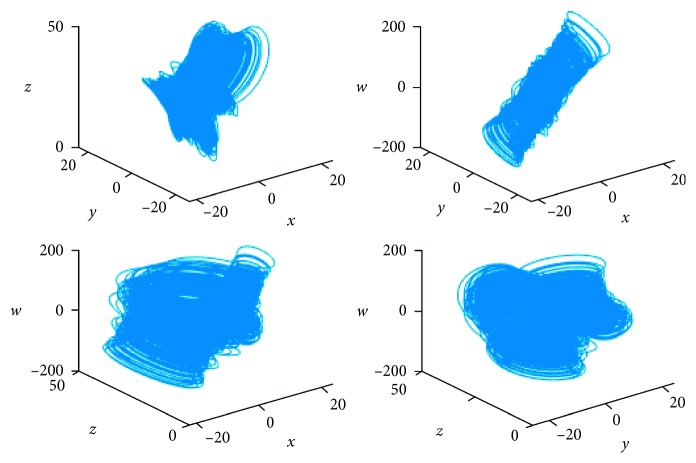
The phase diagram of the Lorenz hyperchaotic system.

Chaotic systems are widely used in the information encryption field because their initial values and parameters are sensitive and pseudorandom [28, 29]. Low-dimensional chaotic systems have small key spaces and weak pseudorandomness. Therefore, many scholars have improved upon low-dimensional chaotic systems by developing chaotic systems to higher dimensions. These improved high-dimensional chaotic systems are called hyperchaotic systems. To generate the four pseudorandom sequences that are required by the encryption algorithm, we apply the Lorenz hyperchaotic system [30] to the encryption algorithm. The Lorenz hyperchaotic system is described as

(1) $$\begin{array}{c} \left\{\begin{array}{c} \dot{x}=a\left(y-x\right)+w,\\ \dot{y}=cx-y-xz,\\ \dot{z}=xy-bz,\\ \dot{w}=-yz+rw, \end{array}\right. \end{array}$$

where *a*, *b*, *c*, and *r* are the four parameters of the Lorenz hyperchaotic system. When *a* = 10, *b* = 8/3, *c* = 28, and −1.52 ≤ *r* ≤ 0.06, the Lorenz hyperchaotic system is in a hyperchaotic state. The hyperchaotic system is iterated by using the Runge–Kutta method when *r* = −1. The simulation results of the Lorenz hyperchaotic system are shown in Figure 1.

### 2.2. Self-Invertible Matrix Encryption

In 1929, Hill proposed an encryption algorithm that used invertible matrices [31]. The fundamental theory of the algorithm is to use a matrix to convert the plain-text into cipher-text, and the key is the matrix itself. The encryption method is described as

(2) $$\begin{array}{c} C=KM\left(\text{mod}\;\;R\right). \end{array}$$

In formula (2), *M* represents a plain-text matrix, *C* represents a cipher-text matrix, *R* is the plain-text value range (in the image encryption process, *R* = 256), *K* represents an encryption key, and matrix *K* must be an invertible matrix. The Hill encryption algorithm is uncompressed. Assuming that the length of the plain-text and cipher-text is *l*, the encryption formula can also be described as

(3) $$\begin{array}{c} \left\{\begin{array}{c} c_{1}=k_{11}m_{1}+k_{12}m_{2}+\ldots +k_{1l}m_{l}\left(\mathrm{mod}\;\;R\right),\\ c_{2}=k_{21}m_{1}+k_{22}m_{2}+\ldots +k_{2l}m_{l}\left(\mathrm{mod}\;\;R\right),\\ \ldots \ldots \\ c_{l}=k_{l1}m_{1}+k_{l2}m_{2}+\ldots +k_{ll}m_{l}\left(\mathrm{mod}\;\;R\right). \end{array}\right. \end{array}$$

In formula (3),

(4) $$\begin{array}{c} C=\left[\begin{array}{c} c_{1}\\ \begin{array}{c} c_{2}\\ \ldots \end{array}\\ c_{l} \end{array}\right],\\ M=\left[\begin{array}{c} m_{1}\\ \begin{array}{c} m_{2}\\ \ldots \end{array}\\ m_{l} \end{array}\right],\\ K={\left(k_{ij}\right)}_{l\times l}=\left[\begin{array}{ccc} k_{11} & \ldots & k_{1l}\\ \begin{array}{c} k_{21}\\ \ldots \end{array} & \ldots & \begin{array}{c} k_{2l}\\ \ldots \end{array}\\ k_{l1} & \ldots & k_{ll} \end{array}\right]. \end{array}$$

The decryption process is the inverse of formula (3) and can be described as

(5) $$\begin{array}{c} M=K^{-1}C\left(\mathrm{mod}\;\;R\right). \end{array}$$

To ensure the existence of matrix *K*^−1^, this paper constructs matrix *K* as a 4 × 4 self-reversible matrix so that *K*^−1^*K*(mod  *R*)=*E*. The decryption process can be simplified as

(6) $$\begin{array}{c} M=K^{-1}C\left(\mathrm{mod}\;\;R\right)=KC\left(\mathrm{mod}\;\;R\right). \end{array}$$

The method of calculating a 4 × 4 self-invertible matrix is as follows. When matrix *A* is a self-invertible matrix, *A*^−1^*A*(mod  *R*)=*E*. If $A=\left[\begin{array}{cc} A_{11} & A_{12}\\ A_{21} & A_{22} \end{array}\right]$, *A*_11_,…, *A*_22_ are 2 × 2 matrices, and formula (7) can be derived:

(7) $$\begin{array}{c} AA^{-1}\left(\mathrm{mod}\;\;R\right)=AA\left(\mathrm{mod}\;\;R\right)=\left[\begin{array}{cc} A_{11} & A_{12}\\ A_{21} & A_{22} \end{array}\right]*\left[\begin{array}{cc} A_{11} & A_{12}\\ A_{21} & A_{22} \end{array}\right]\left(\mathrm{mod}\;\;R\right)=E. \end{array}$$

Then, formula (8) can be calculated by expanding formula (7):

(8) $$\begin{array}{c} A_{12}A_{21}=E-A_{11}^{2}=\left(E-A_{11}\right)\left(E+A_{11}\right). \end{array}$$

To construct the self-invertible matrix, *A*_12_ is constructed as a factor of (*E* − *A*11) and *A*_21_ is constructed as a factor of (*E* + *A*_11_). *k* is a prime number that is mutually prime with *R.* When *A*_12_ ≠ 0, formula (9) can be derived:

(9) $$\begin{array}{c} \left\{\begin{array}{c} A_{11}=-A_{22}\left(\mathrm{mod}\;\;R\right),\\ A_{12}=k\left(E-A_{11}\right)\left(\mathrm{mod}\;\;R\right),\\ A_{21}=\frac{\left(E+A_{11}\right)}{k}\left(\mathrm{mod}\;\;R\right). \end{array}\right. \end{array}$$

The self-invertible matrix *A* can be calculated using a given matrix *A*_22_. Taking *k* = 3, *R* = 256 and $A_{22}=\left[\begin{array}{cc} 215 & 52\\ 20 & 45 \end{array}\right]$as an example, to calculate the self-reversible matrix *A*, first we should calculate the matrix *A*_11_. Because *A*_11_ = −*A*_22_ (mod *R*), we can get

(10) $$\begin{array}{c} A_{11}=\left[\begin{array}{cc} -215 & -52\\ -20 & -45 \end{array}\right]\left(\mathrm{mod}\;\;256\right)=\left[\begin{array}{cc} 41 & 204\\ 236 & 211 \end{array}\right]. \end{array}$$

Because *A*_12_ = *k*(*E* − *A*_11_)(mod *R*), we can get

(11) $$\begin{array}{c} A_{12}=3\;*\;\left(E-A_{11}\right)\left(\mathrm{mod}\;\;256\right)=\left[\begin{array}{cc} 136 & 156\\ 60 & 138 \end{array}\right]. \end{array}$$

Because *A*_21_ = (*E* + *A*_11_)/*k*(mod *R*), we can get

(12) $$\begin{array}{c} A_{21}=\frac{\left(E+A_{11}\right)}{3}\left(\mathrm{mod}\;\;256\right)=\left[\begin{array}{cc} 14 & 68\\ 164 & 156 \end{array}\right]. \end{array}$$

Then, the self-invertible matrix *A* can be obtained. $A=\left[\begin{array}{cccc} 41 & 204 & 136 & 156\\ 236 & 211 & 60 & 138\\ 14 & 68 & 215 & 52\\ 164 & 156 & 20 & 45 \end{array}\right]$. It can be verified that *A*^−1^*A*(mod  256)=*E*.

### 2.3. Fractal

In 1967, Mandelbrot published a paper entitled, “How Long is the British Coastline,” in Science. In it, he used fractals to describe a large class of complex irregularities that cannot be described using traditional Euclidean geometry in nature. It marked the emergence of fractal thought. A fractal is a set of mathematical theories that uses fractal features as the research object. Some common geometric fractals are the Koch curve, the H-fractal, the Sierpinski triangle, and the Vivsek triangle. Fractal theory is not only a frontier and important branch of nonlinear science but also a new cross-discipline. It is a new mathematics discipline that studies the characteristics of a class of phenomena. Compared with its geometric form, it is more connected with differential equations and dynamic systems theory. The fractals are not limited to geometric forms and times and processes can also form fractals. As a new concept and method, the fractal is being applied in many fields. In recent years, fractal sensitivity, especially the sensitivity of the Mandelbrot sets and Julia sets to initial values, has been widely used in image encryption.

**Figure 2:**
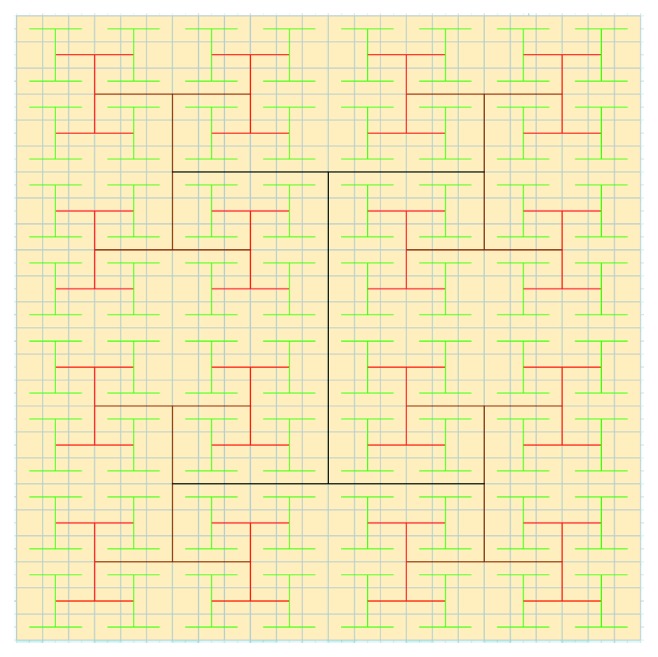
The diagram of the H-fractal.

The H-fractal is a kind of fractal, and the diagram of the H-fractal is shown in Figure 2. Fractal graphics can be used for information encryption and security. This algorithm uses the H-fractal to encrypt image information.

## 3. Encryption Scheme

### 3.1. Key Generation

SHA-3 algorithm is a kind of Secure Hash Algorithm. This encryption algorithm uses the Hash sequence that is generated by the SHA-3(256) algorithm, and the prime number *k* is used to construct the self-invertible matrices that are used as keys. The initial values *x*_0_, *y*_0_, *z*_0_, and *w*_0_ of the Lorenz hyperchaotic system are generated by the original image. To obtain the 256 bit binary Hash sequence H, the algorithm inputs the original image into the SHA-3(256) function. Then, the sequence H is divided into 32 8 bit binary sequences as *h*_1_, *h*_2_,…, *h*_32_, and the initial values of the Lorenz hyperchaotic system are calculated using

(13) $$\begin{array}{c} \left\{\begin{array}{c} x_{0}=\frac{\left(h_{1}\;⊕\;h_{2}\;⊕\;h_{3}\;⊕\;h_{4}\;⊕\;h_{5}\;⊕\;h_{6}\;⊕\;h_{7}\;⊕\;h_{8}\right)}{256}+x_{0}^{\prime },\\ y_{0}=\frac{\left(h_{9}\;⊕\;h_{10}\;⊕\;h_{11}\;⊕\;h_{12}\;⊕\;h_{13}\;⊕\;h_{14}\;⊕\;h_{15}\;⊕\;h_{16}\right)}{256}+y_{0}^{\prime },\\ z_{0}=\frac{\left(h_{17}\;⊕\;h_{18}\;⊕\;h_{19}\;⊕\;h_{20}\;⊕\;h_{21}\;⊕\;h_{22}\;⊕\;h_{23}\;⊕\;h_{24}\right)}{256}+z_{0}^{\prime },\\ w_{0}=\frac{\left(h_{25}\;⊕\;h_{26}\;⊕\;h_{27}\;⊕\;h_{28}\;⊕\;h_{29}\;⊕\;h_{30}\;⊕\;h_{31}\;⊕\;h_{32}\right)}{256}+w_{0}^{\prime }. \end{array}\right. \end{array}$$

The number of iterations of the hyperchaotic system is selected according to the size of the original image after obtaining the initial values of the Lorenz hyperchaotic system. If the size of the original image is *M* × *N*, it is necessary to iterate the Lorenz hyperchaotic system *M* × *N* + 800 times and delete the first 800 iterations to avoid the transient effect. Finally, four pseudorandom sequences *X*, *Y*, *Z*, and *W* of length *M* × *N* are obtained.

### 3.2. Scrambling Based on the Self-Invertible Matrix

The sequence *Y* that is generated by the Lorenz hyperchaotic system is chosen to produce the self-invertible matrices. When an *M* × *N* original image is encrypted using dynamic self-invertible matrices, the encryption process is described as follows:

  Step 1: The original image is divided into *M* × *N*/16 matrices that are sized 4 × 4, which are, respectively, labeled as *PM*_*i*_ (*i* = 1, 2,…, *M* × *N*/16) using the row-first method.
  Step 2: The pseudorandom sequence *Y* in the Lorenz chaotic system is conducted using formula (14) to obtain the pseudorandom matrix *YM.* In formula (14), floor(*x*) is a floor function, and reshape(*x*) is a column-first ordering function:

(14) $$\begin{array}{c} YM=\text{reshape}\left(\left(\mathrm{mod}\left(\text{floor}\left(\left(Y\left(:\right)\;*\;10^{2}-\text{floor}\left(Y\left(:\right)*\;10^{2}\right)\right)\;*\;10^{10}\right),256\right),256,256\right)\right). \end{array}$$

  Step 3: The matrix *YM* is divided into *M* × *N*/16 matrices that are sized 4 × 4 according to the method in Step 1, and we label the 4 × 4 matrices as *YM*_*i*_ (*i* = 1, 2,…, *M* × *N*/16).
  Step 4: The 4 × 4 matrices *YM*_*i*_ (*i* = 1, 2,…, *M* × *N*/16) are divided into four 2 × 2 matrices, and the matrices in the upper left corner are reserved as *YM*_*i*_′ (*i* = 1, 2,…, *M* × *N*/16).
  Step 5: The 2 × 2 matrices *YM*_*i*_′ (*i* = 1, 2,…, *M* × *N*/16) are transformed into the self-invertible matrices as *K*_*i*_ (*i* = 1, 2,…, *M* × *N*/16) using the prime number *k* with the construction method of the self-invertible matrix.
  Step 6: The cipher matrices *C*_*i*_ (*i* = 1, 2,…, *M* × *N*/16) are calculated, and *C*_*i*_ = *K*_*i*_*PM*_*i*_ (*i* = 1, 2, 3,…, *M* × *N*/16).
  Step 7: The cipher image is composed of the cipher matrices *C*_*i*_ (*i* = 1, 2,…, *M* × *N*/16) using the row-first method.

The decryption process of the self-invertible matrix encryption algorithm is the inverse of the encryption process, so it will not be described again.

### 3.3. H-Fractal Diffusion

**Figure 3:**
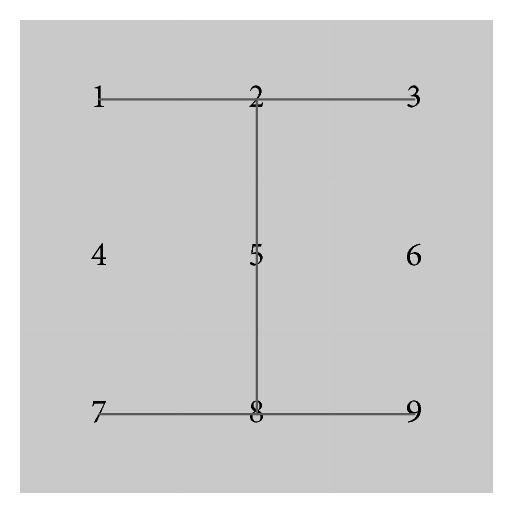
The diagram of H-fractal diffusion.

The H-fractal cross-diffusion method that is proposed in this paper uses the intermediate pixel that is covered by the H-fractal as an operator to cross-process the two pixels on both ends of the H-fractal to complete the diffusion. Taking a 3 × 3 block as an example, the diffusion process based on the H-fractal is shown in Figure 3.

**Figure 4:**
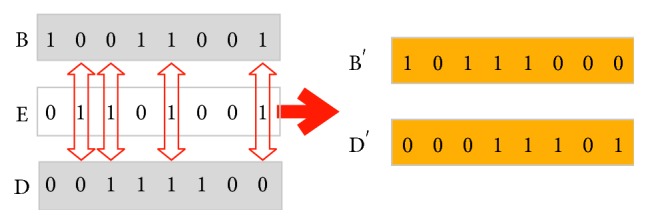
The diagram of the crossover operation.

In Figure 3, pixel 2 is used as a control word, and pixel 1 and pixel 3 are controlled to perform a crossover operation. Then, pixel 8 is used as a control word, and pixel 7 and pixel 9 are controlled to perform a crossover operation. Finally, pixel 5 is used as a control word, and pixel 2 and pixel 8 are controlled to perform a crossover operation. The crossover operation method is shown in Figure 4, where *E* is the control word, *B* and *D* are the endpoint pixels, and *B*′ and *D*′ are the pixels after the crossover operation. When the values of the binary control bits in the pixel *E* are “1,” the binary words in pixels *B* and *D* corresponding to the control bits are exchanged. Conversely, when the values of the binary control bits in pixel *E* are “0,” the binary words in pixels *B* and *D* corresponding to the control bits have no operation. The decryption process is the inverse of the diffusion process, so it will not be described here.

**Figure 5:**
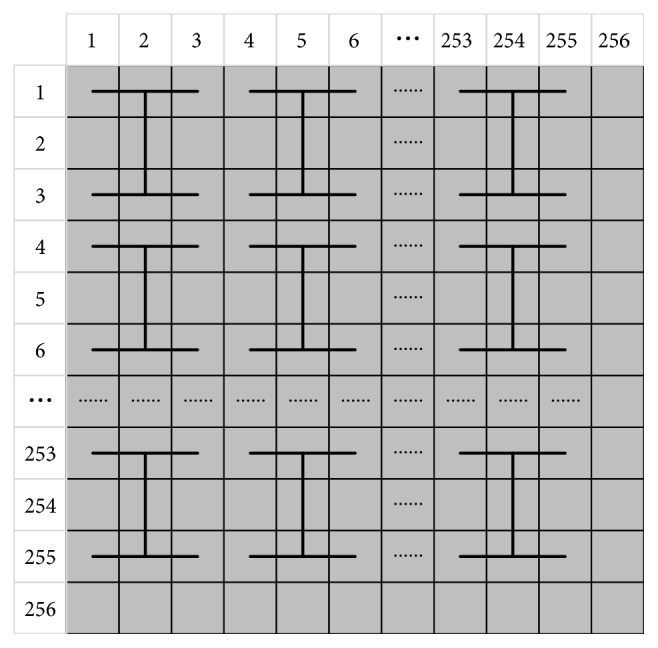
The image covered by the H-fractal.

Taking a 256 × 256 image as an example, the image that is covered by the H-fractal is shown in Figure 5. The first pixel in the upper left corner of the image is used as the starting point to construct the H-fractal. The pixels that are not covered by the H-fractal in the image are not operated on.

### 3.4. Cipher-Pixels Feedback Encryption

In this paper, a cipher-pixel feedback method is used to enhance the diffusion effect. The cipher-pixel feedback method makes the pixels in the front of the pixel sequence affect the pixels behind them. Assuming that the size of the original image is *M* × *N*, the specific process of cipher-pixel feedback is described as follows. First, rearrange the original images into a pixel sequence *P*{1, 2, 3,…, *M* × *N*}. Then, a diffused sequence *P*′{1, 2, 3,…, *M* × *N*} is obtained by operating on sequence *P* using

(15) $$\begin{array}{c} \left\{\begin{array}{c} P^{\prime }\left(1\right)=P\left(1\right),\\ P^{\prime }\left(i\right)=\text{bitxor}\left(P\left(i\right),P^{\prime }\left(i-1\right)\right),\;2\leq i\leq M\times N. \end{array}\right. \end{array}$$

### 3.5. Scrambling Process

The algorithm uses the pseudorandom sequences *X*, *Z*, and *W* that are generated by the Lorenz hyperchaotic system to scramble the image. Taking the *M* × *N* image as an example, assuming that the given key is a pseudorandom sequence *S*{1, 2, 3,…, *M* × *N*}, the global scrambling operation is described as follows. First, the original image is expanded into a one-dimension pixel sequence *P*_1_{1, 2, 3,…, *M* × *N*}, and the positions of the pixels in sequence *P*_1_ are corresponded with the positions of the elements in sequence *S*. Then, the pseudorandom sequence *S* is rearranged in ascending order to obtain an index sequence *S*′. Finally, the pixel sequence *P*_1_{1, 2, 3,…, *M* × *N*} is mapped to the new pixel sequence *P*_1_′{1, 2, 3,…, *M* × *N*} according to the rules for mapping the elements in the sequence *S* to the sequence *S*′. The decryption process of scrambling is the inverse of the encryption process, so it will not be described here.

### 3.6. Encryption Scheme

**Figure 6:**
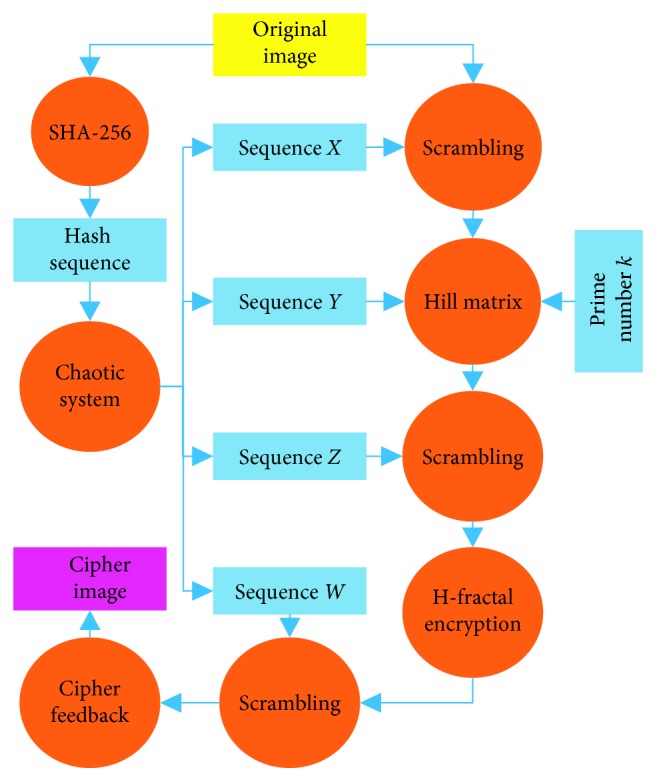
The flow chart of the encryption scheme.

The flow chart of the encryption scheme is shown in Figure 6. Taking image *I* that is sized 256 × 256 as an example, the encryption process is described as follows:

  Step 1: Image **I** is input into the SHA-3(256) algorithm to obtain a Hash sequence *H*.
  Step 2: The initial values *x*_0_, *y*_0_, *z*_0_, and *w*_0_ of the Lorenz hyperchaotic system are obtained by conducting the Hash sequence *H*.
  Step 3: The Lorenz hyperchaotic system is iterated *M* × *N* + 800 times, and four sequences *X*, *Y*, *Z*, and *W* are obtained by discarding the values of the first 800 iterations.
  Step 4: The image matrix **I**_1_ is obtained by scrambling the original image **I** using sequence *X.*
  Step 5: The image matrix **I**_2_ is obtained by using the dynamic self-invertible matrix encryption method to encrypt image matrix **I**_1_ using sequence *Y.*
  Step 6: The image matrix **I**_3_ is obtained by scrambling the image matrix **I**_2_ using sequence *Z.*
  Step 7: The image matrix **I**_4_ is obtained by using the H-fractal encryption method to encrypt image matrix **I**_3_.
  Step 8: The image matrix **I**_5_ is obtained by scrambling the image matrix **I**_4_ using sequence *W.*
  Step 9: The cipher-text feedback operation is performed on the image matrix **I**_5_ to obtain the image matrix **I**_6_, namely, the ciphertext image.

The decryption process of this encryption scheme is the inverse of the encryption process, so it will not be repeated.

## 4. Simulation Results and Security Analysis

**Figure 7:**
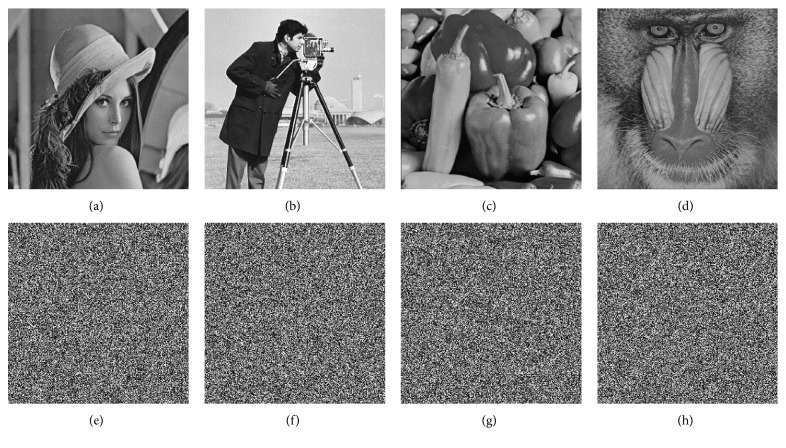
The original images and their cipher images. (a) Original Lena image. (b) Original Cameraman image. (c) Original Pepper image. (d) Original Baboon image. (e) Cipher Lena image. (f) Cipher Cameraman image. (g) Cipher Pepper image. (h) Cipher Baboon image.

In order to verify the effectiveness and feasibility of our algorithm, simulated experiments are undertaken on the MATLAB R2018a platform. The environment of development is Windows 7, 4.00 GB RAM, Intel(R) Core(TM) i3-4130 CPU @ 3.4 GHz. Mean execution time of test images with size of 256 × 256 is 1.518s. Part of the encryption keys are set as *x*_0_′=0, *y*_0_′=0, *z*_0_′=0, *w*_0_′=0, *a*=10, *b*=8/3, *c*=28, *r*=−1 and the Hash sequences are generated by the original images. Some original images are shown in Figures 7(a)–7(d), and their cipher images are shown in Figures 7(e)–7(h). Because the encryption algorithm that we proposed is lossless, the decrypted images of the cipher images are exactly the same. The algorithm does not destroy the characteristics of the original image.

### 4.1. Key Sensitivity Analysis

**Figure 8:**
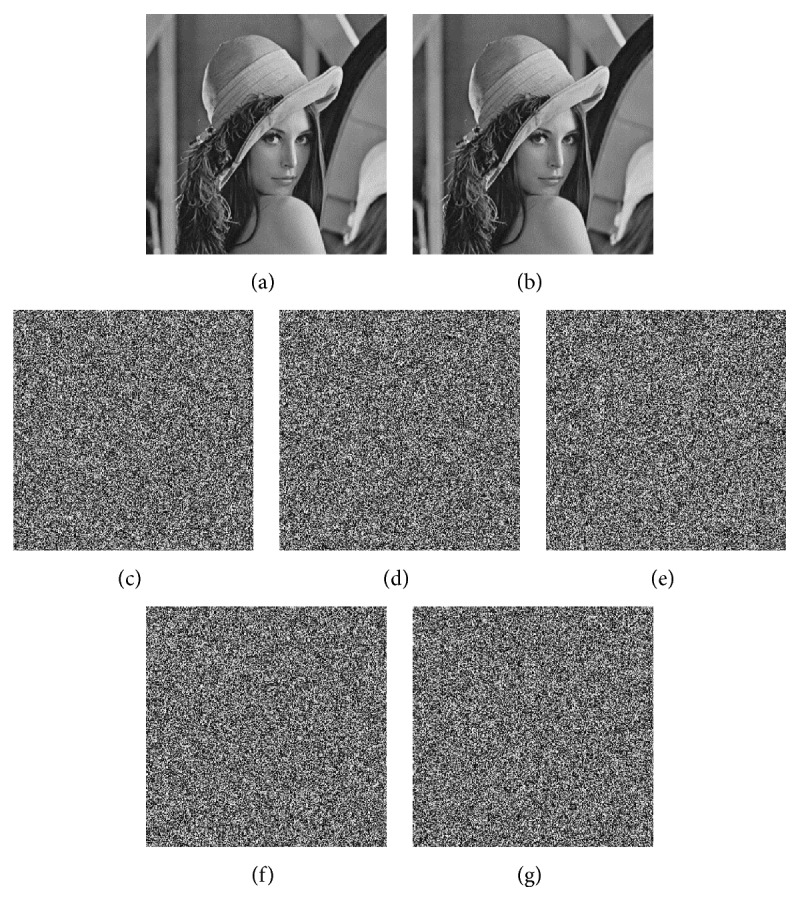
The correct decrypted image and the incorrectly decrypted images due to a slight change in the initial values of the Lorenz hyperchaotic system. (a) The original Lena image. (b) The correct decrypted image. The decrypted image (c) when *x*_0_ is changed by 10^−13^, (d) when *y*_0_ is changed by 10^−13^, (e) when *z*_0_ is changed by 10^−13^, (f) when *w*_0_ is changed by 10^−13^, and (g) when *k* = 5.

The encryption algorithm that is proposed in this paper uses the 256 bit Hash sequence that is generated by the SHA-3(256) algorithm and the prime number *k* as the key. The key space of the 256 bit Hash sequence is 2^128^. Therefore, the key space of the algorithm is large enough to resist brute-force attacks. The initial values of the Lorenz hyperchaotic system are generated by the Hash sequence. When the Hash sequence has a slight change, the initial value of the hyperchaotic system also changes. The algorithm is very sensitive to the changes of these initial values. When these initial values have been slightly changed by 10^−13^, the encryption system cannot be decrypted. When the prime number *k* = 3, the original image and the correct decrypted image are shown in Figures 8(a) and 8(b). In Figures 8(c)–8(g), the decrypted images after the initial values have been changed are listed. The encryption algorithm is very sensitive to the key, and the algorithm is sufficient to resist attacks on the key.

### 4.2. Differential Attack Analysis

When the original image has a slight change, the cipher image will have a big change. This phenomenon reflects that the encryption system is very sensitive to changes in the original image. The higher the sensitivity of the plaintext, the stronger the cryptosystem's ability to resist differential attacks. Here, we use the number of pixel changes rate (NPCR) and the unified average changing intensity (UACI) to measure the antidifferential attack capability of the encryption system. The methods for calculating the NPCR and UACI are described as

(16) $$\begin{array}{c} \left\{\begin{array}{c} \text{NPCR}=\frac{\sum_{i,j}\left|Sign\left(P_{1}\left(i,j\right)-P_{2}\left(i,j\right)\right)\right|}{M\times N}\times 100\%,\\ \text{UACI}=\frac{\sum_{i,j}\left|P_{1}\left(i,j\right)-P_{2}\left(i,j\right)\right|}{255\times M\times N}\times 100\%. \end{array}\right. \end{array}$$

In formula (15), *P*_1_ is the correct cipher image, and *P*_2_ is the cipher image where the original image has a little change. *M* and *N*, respectively, represent the length and width of the image. Sign(*x*) represents the symbol function, and its calculation method is described as

(17) $$\begin{array}{c} \text{Sign}\left(x\right)=\;\;\left\{\begin{array}{c} \;\;1,\;\;\;x>0,\;\;\\ \;\;0,\;\;\;x=0,\\ -1,\;\;\;x<0. \end{array}\right. \end{array}$$

The maximum theoretical value of the NPCR is 100%, and the ideal value of the UACI is 33.4635%. The larger the NPCR is, the greater the pixel changes. When the original image has been changed by 1 bit, the values of the NPCR and UACI are shown in Table 1. In addition, the values of the NPCR and UACI in the references [32] are listed in Table 1. By comparison, it is known that the algorithm that we proposed is very sensitive to plaintext and can resist differential attacks very well.

### 4.3. Information Entropy Analysis

Information entropy is the concept that was proposed by Shannon to quantify information. It can usually be expressed as *H*(*s*). The concept of information entropy is described as

(18) $$\begin{array}{c} H\left(s\right)=-\overset{n}{\underset{i=1}{\sum }}p\left(i\right)\log_{2}\;p\left(i\right). \end{array}$$

In formula (17), *p*(*i*) represents the probability of the occurrence of the case and *n* represents the total number of all possible occurrences. The information entropy is used to measure the randomness of the information. The closer the information entropy is to the ideal value, the stronger the randomness of the information is. The pixels in the grayscale image are all in the interval [0, 255]. When the image is completely random, the probability of each pixel value is 1/256, so the information entropy of a completely random grayscale image is 8. The information entropies of some original images and their cipher images are listed in Table 2. It can be seen from the comparison that the cipher images that are encrypted by this algorithm are close to random.

### 4.4. Histogram Statistical Analysis

**Figure 9:**
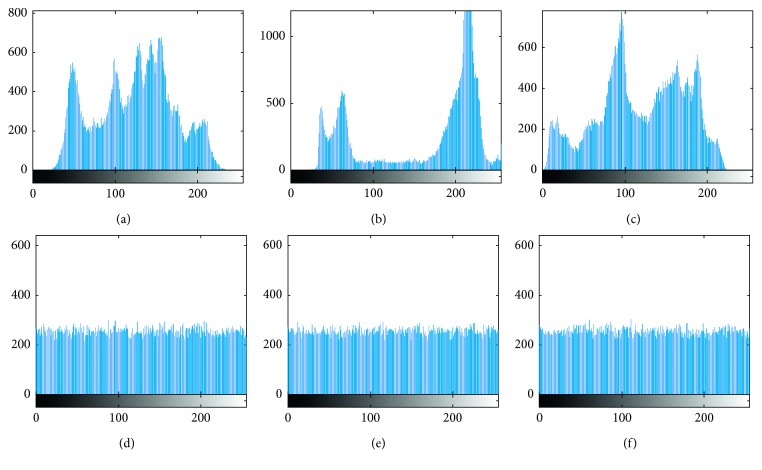
The histograms of the original images and their cipher images. The histograms of (a) the Lena image, (b) the Cameraman image, (c) the Pepper image, (d) the Lena cipher image, (e) the Cameraman cipher image, and (f) the Pepper cipher image.

Histogram statistical analysis is a kind of statistical attack, and the histogram can characterize the image. The pixel distribution in the histogram of the original image is not uniform, which is not conducive to resisting statistical attacks. A good encryption algorithm can make the pixel distribution in the histogram of the cipher image more uniform, and thus, it can resist known-plaintext attacks and chosen-plaintext attacks. The histograms of the original images are shown in Figures 9(a)–9(c), and the histograms of the cipher images are shown in Figures 9(d)–9(f). It can be seen from the comparison that the algorithm can destroy the histogram of the statistical law of the original image and achieve good performance.

### 4.5. Correlation Analysis

**Figure 10:**
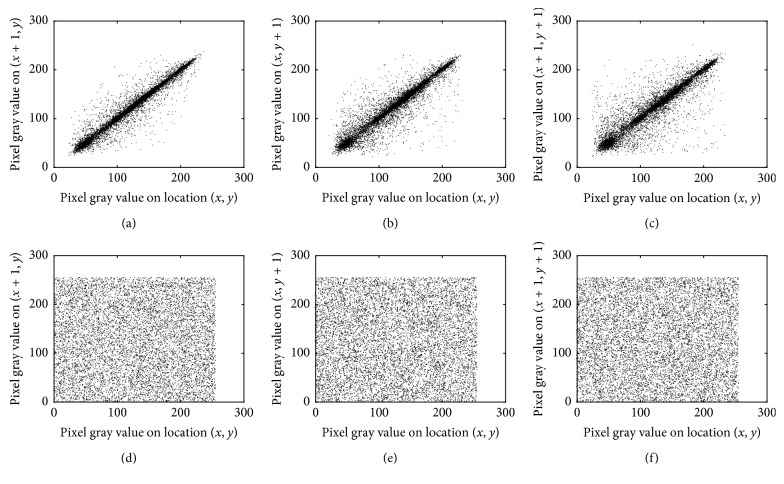
The values of the selected pixels and their adjacent pixels in different directions. Horizontal correlation of (a) the original image and (d) the cipher image. Vertical correlation of (b) the original image and (e) the cipher image. Diagonal correlation of (c) the original image and (f) of the cipher image.

10000 pixels and their adjacent pixels from the original Lena image are randomly selected in the horizontal, vertical, and diagonal directions, and the values of these pixels are shown in Figures 10(a)–10(c), respectively. It can be seen from the analysis that there is a strong correlation between adjacent pixels. A good encryption algorithm can break the correlation between adjacent pixels, and it can enhance the ability to resist statistical attacks. 10000 pixels and their adjacent pixels from the cipher Lena image are randomly selected in the horizontal, vertical, and diagonal directions, and the values of these pixels are shown in Figures 10(d)–10(f), respectively. It can be seen from the comparison that the algorithm can break the correlation between adjacent pixels.

The correlation coefficient is used as an indicator to measure the correlation between adjacent pixels. Its calculation method is described as

(19) $$\begin{array}{c} \left\{\begin{array}{c} E\left(x\right)=\frac{1}{N}\overset{N}{\underset{i=1}{\sum }}x_{i},\\ D\left(x\right)=\frac{1}{N}\overset{N}{\underset{i=1}{\sum }}{\left(x_{i}-E\left(x\right)\right)}^{2},\\ \mathrm{cov}\left(x,y\right)=\frac{1}{N}\overset{N}{\underset{i=1}{\sum }}\left(x_{i}-E\left(x\right)\right)\left(y_{i}-E\left(y\right)\right),\\ r_{xy}=\frac{\mathrm{cov}\left(x,y\right)}{\sqrt{D\left(x\right)}\sqrt{D\left(y\right)}}. \end{array}\right. \end{array}$$

In formula (18), *N* is the total number of selected pixels, *E*(*x*) is the mean of the selected pixels, *D*(*x*) represents the variance of the selected pixels, cov(*x*, *y*) represents the covariance of the selected pixels, and *r* represents the correlation coefficient. An absolute value of the correlation coefficient that is close to 1 indicates that the correlation of the data is strong, and an absolute value of the correlation coefficient that is close to 0 indicates that the data have almost no correlation. The correlation coefficients of the original images and the cipher images are listed in Table 3. It can be seen from the comparison that the image correlation coefficient of the encryption algorithm is almost zero, and the encryption algorithm can break the correlation between adjacent pixels.

### 4.6. Antiocclusion Attack Capability Analysis

**Figure 11:**
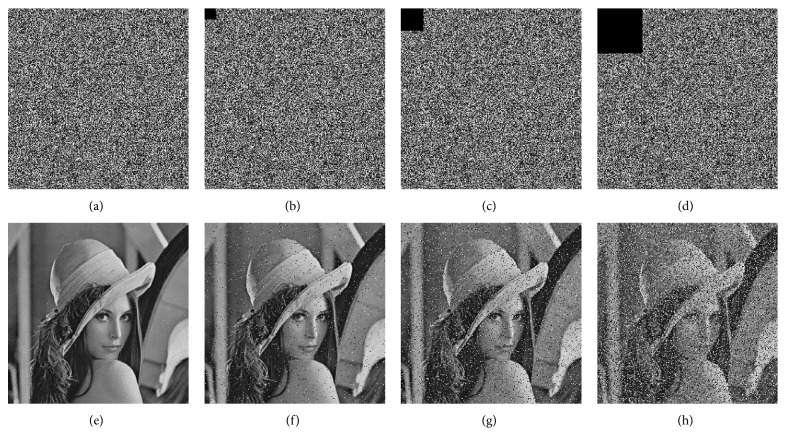
The reduced cipher images and their corresponding decrypted images. (a) Correct cipher image. (b) 1/256 occlusion. (c) 1/64 occlusion. (d) 1/16 occlusion. (e) The correct decrypted image. Decrypted image (f) with 1/256 occlusion, (g) with 1/64 occlusion, and (h) with 1/16 occlusion.

The antiocclusion attack capability of the encryption system can reflect the degree of recovery of the decrypted image when the cipher image data are lost. In a cryptosystem without global scrambling, when the cipher image data are lost, its decrypted image may lose some important features in the original image. The Lena cipher images cut by 0, 1/256, 1/64, and 1/16 are shown in Figures 11(a)–11(d), and the corresponding decrypted images are shown in Figures 11(e)–11(h).

The NPCR, UACI, and correlation coefficients between the original images and the decrypted images after the occlusion attacks are listed in Table 4. The comparison between the data proves that the encryption algorithm that we proposed has a good antiocclusion attack capability.

### 4.7. Practicality Analysis

Some characteristics of the cipher images with different sizes encrypted by the proposed algorithm are listed in Table 5; these images are encrypted by *k* = 3. It can be seen from the data in Table 5 that the proposed algorithm can encrypt images with different size and has good encryption effects.

## 5. Conclusions

In this paper, an image encryption algorithm based on the H-fractal structure and dynamic self-invertible matrix is proposed. The algorithm uses the Hash sequence that is generated by the SHA-3(256) algorithm and a prime number as the keys. The image is scrambled and diffused by the four pseudorandom sequences that are generated by the Lorenz hyperchaotic system. In this encryption scheme, a cross-diffusion operation based on the H-fractal structure is applied for the first time. The algorithm enriches the means of digital image encryption. It has high security to resist brute-force attacks and statistical attacks, and it has the ability to recover when the cipher data are lost. Thus, this algorithm can be used to protect the security of digital images.

## Figures and Tables

**Table 1:** NPCR and UACI.

Image	The proposed scheme		Reference [32]		Reference [33]	
	NPCR (%)	UACI (%)	NPCR (%)	UACI (%)	NPCR (%)	UACI (%)
Lena	99.6292	33.4481	99.5986	33.4561	99.58	33.08
Cameraman	99.6094	33.6017	99.5590	33.4439	99.90	33.15
Peppers	99.6216	33.5715	99.5803	33.4324	99.71	32.19
Baboon	99.6140	33.5152	—	—	99.59	31.56

**Table 2:** The information entropies of some original images and their cipher images.

Image	Entropy					
	Original	*k* = 3	*k* = 37	*k* = 487	Reference [32]	Reference [33]
Lena	7.4532	7.9971	7.9974	7.9974	7.9971	7.9968
Cameraman	6.9046	7.9976	7.9971	7.9972	7.9971	7.9904
Peppers	7.5797	7.9973	7.9978	7.9969	7.9968	7.9961
Baboon	7.0092	7.9972	7.9973	7.9975	—	7.9971

**Table 3:** The correlation coefficients in different directions.

Image	Original image			Cipher image		
	Horizontal	Vertical	Diagonal	Horizontal	Vertical	Diagonal
Lena	0.9680	0.9349	0.9069	0.0078	0.0040	−0.0050
Cameraman	0.9467	0.9180	0.9054	−0.0019	−0.0051	0.0032
Peppers	0.9731	0.9664	0.9381	0.0051	0.0037	0.0014
Baboon	0.8327	0.8759	0.7890	−0.0065	−0.0038	0.0065

**Table 4:** The NPCRs, UACIs, and correlation coefficients of the images after the occlusion attack.

Occlusion	NPCR	UACI	Correlation coefficients		
			Horizontal	Vertical	Diagonal
0	0	0	0.9680	0.9349	0.9069
1/256	3.5339	1.0733	0.8371	0.8222	0.7957
1/64	12.2253	3.6316	0.6419	0.6057	0.5811
1/16	35.9364	10.6799	0.2669	0.2516	0.2327

**Table 5:** Analysis of cipher images with different sizes.

Cipher images	Correlation Coefficients			Entropies	NPCR (%)	UACI (%)
	Horizontal	Vertical	Diagonal			
Lena 128 × 128	0.0002	−0.0008	−0.0028	7.9882	99.6094	33.1566
Lena 512 × 512	−0.0061	0.0014	−0.0012	7.9994	99.6147	33.4730
Cameraman 128 × 128	0.0028	0.0020	0.0011	7.9878	99.6399	33.3091
Cameraman 512 × 512	0.0012	−0.0052	−0.0028	7.9993	99.5831	33.4335
Peppers 128 × 128	0.0028	−0.0078	0.0098	7.9887	99.6216	33.3656
Peppers 512 × 512	−0.0005	0.0018	0.0063	7.9992	99.5899	33.4390
Baboon 128 × 128	−0.0062	0.0012	0.0062	7.9869	99.7253	33.6223
Baboon 512 × 512	−0.0071	−0.0053	−0.0067	7.9993	99.6162	33.5096

## Data Availability

The data used to support the findings of this study are available from the corresponding author upon request.
